# Newer antipsychotics and the rabbit syndrome

**DOI:** 10.1186/1745-0179-3-6

**Published:** 2007-06-11

**Authors:** Mario Catena Dell'Osso, Andrea Fagiolini, Francesca Ducci, Azadeh Masalehdan, Antonio Ciapparelli, Ellen Frank

**Affiliations:** 1 Dipartimento di Scienze Neurologiche e Psichiatriche, University of Florence, Florence, Italy; 2 Dipartimento di Psichiatria, Neurobiologia, Farmacologia e Biotecnologie, University of Pisa, Pisa, Italy; 3 Department of Psychiatry, University of Pittsburgh School of Medicine, Western Psychiatric Institute and Clinic, Pittsburgh, Pennsylvania, USA; 4 National Institutes of Health, Bethesda, USA

## Abstract

**Background:**

Rabbit syndrome is a movement disorder that is associated with long-term exposure to neuroleptic medications. Of particular interest and importance is the risk of rabbit syndrome with exposure to the newer atypical antipsychotics. Our recent experience with such a case brought to light the importance of exploring this risk.

**Methods:**

MEDLINE and PubMed (1972–2006) databases were searched for English language articles using the keywords rabbit syndrome, tardive dyskinesia, antipsychotic, extrapyramidal symptoms and side effects. A recent case study is used to expand upon the literature available on newer antipsychotics and rabbit syndrome.

**Results:**

We reviewed papers that addressed the following aspects of rabbit syndrome 1) the clinical manifestations 2) prevalence and risk factors, 3) etiopathogenesis 4) older antipsychotics and rabbit syndrome 5) newer antipsychotics, 6) treatment options. Moreover, we report a case of RS in a 50 year old white female, diagnosed with bipolar I disorder, that, after the discontinuation of risperidone, developed involuntary movements of the mouth that were fine, rhythmic and rapid, along the vertical axis, and without involvement of the tongue. After the re-introduction of risperidone, the symptoms decreased in a few hours and disappeared after 3 days.

**Conclusion:**

Eleven cases of rabbit syndrome have been documented since the implementation of newer antipsychotics. Future research is needed to better understand the etiopathogenesis of rabbit syndrome in psychiatric populations treated with the atypical antipsychotics. Understanding the differences and similarities of rabbit syndrome and tardive dyskinesia is crucial to the creation of a successful treatment paradigm.

## Background

Rabbit syndrome (RS) is characterized by fine, rapid, rhythmic movements along the vertical axis of the mouth. Long-term exposure to the older neuroleptics has clearly been associated with RS, but little is known regarding the risk of RS due to the exposure to the newer atypical antipsychotics. This syndrome is considered a distinct neuroleptic-induced extrapyramidal syndrome. There is evidence that RS combines features of both Parkinson's disease and tardive dyskinesia [1-4].

In the present study we review the current knowledge on the clinical manifestations, the etiopathogenesis, and the prevalence of the RS. We also report on a patient who developed the syndrome after discontinuing an atypical antipsychotic, comment on the few similar cases that have been recently published, and review the possible treatment options and the outcome of patients developing this syndrome.

## Methods

MEDLINE and PubMed (1972–2006) databases were searched for English language articles using the keywords rabbit syndrome, tardive dyskinesia, antipsychotic, extrapyramidal symptoms. We also performed a chart review for the case of RS that occurred in one of our patients upon discontinuation of an atypical neuroleptic.

## Results and discussion

### Clinical manifestations

RS is characterized by fine, rapid, rhythmic movements along the vertical axis of the mouth. These involuntary movements occur at a frequency of approximately 5 Hz. The oral movements are often accompanied by a popping sound that is produced by the rapid smacking on one's lips. This syndrome is limited exclusively to the territory of the oral and masticatory muscles and does not involve the tongue [1]. The pattern of movement contrasts tardive dyskinesia (TD), another form of oral dyskinesia, in which the tongue is involved in making slower and less regular movements.

Similar to TD, the movements of RS increase in situations of fatigue and anxiety. Stressful situations may also be a trigger for intensifying the symptoms of RS. RS always worsens during attention, concentration, and motor performances. In these situations, RS is accompanied by an increase in muscle tone. Jus et al. [5] found that when patients were asked to follow a labyrinth pattern with a pencil, RS symptoms intensified. Different from other types of oral dyskinesias such as buccolingual and buccolinguo-masticatory syndromes, RS cannot be suppressed voluntarily by the patient [6]. RS can be associated with drug-induced parkinsonism [7,8] and TD [3,9]. RS symptoms are similar to Parkinson's symptoms in their persistence during stage 1 non-REM sleep, whereas a cessation of symptoms is observed in the same situation with TD.

### Prevalence and risk factors

RS is believed to be a rare condition affecting only a small fraction of the psychiatric population using neuroleptics. Yassa and Lal [8] carried out the first prevalence study by focusing on the inpatient population of a psychiatric institution and reported a 2.3% prevalence rate of RS in a sample of 266 inpatients receiving older neuroleptics, either as monotherapy or in combination with anticholinergic agents. The prevalence rate of RS in patients solely taking neuroleptics was 4.4%. Another prevalence study [10] found a rate of 1.5% for RS among neuroleptic-treated patients in a geriatric mental health clinic. To the best of our knowledge, no prevalence study has been published to date on the relationship between RS and atypical antipsychotics.

Gender and age are also believed to be related to the development of RS. This syndrome is found predominantly in middle-aged and elderly patient populations [11]; women are also believed to be at higher risk for developing RS than their male counterparts [7]. Data from Tables 1 and 2 indicates that most of the cases of RS reported to-date have involved patients with a diagnosis of schizophrenia. Previous brain damage has also been associated with RS [1].

**Table 1:** Older Antipsychotics and the Rabbit Syndrome.

Patient	Age	Gender	Diagnosis	Duration of previous antipsychotic treatment	Drug (mg)	Parkinsonian Syntoms	Tardive Dyschinesia
Patient 1 [1]	66	F	Schizophrenia	Several years	Perphenazine (8)	No	No
Patient 2 [1]	67	F	Schizophrenia	Several years	Trifluoperazine (5) Benzotropine	Yes	Yes
Patient 3 [1]	54	F	Schizophrenia	Several years	Clhorpromazine (50) Trifluoperazine (5) Thioridazine (50)	Yes	No
Patient 4 [1]	66	M	Schizophrenia	Several years	Trifluoperazine (5) Methotrimipramine (125) Benzotropine (2)	No	No
Patient 5 [1]	47	M	Schizophrenia	Several years	Thioridazine (100)	Yes	No
Patient 6 [2]	Between 47 and 62	M	-	Several years	-	No	No
Patient 7 [2]	Between 47 and 62	M	-	Several years	-	No	No
Patient 8 [2]	Between 47 and 62	M	-	Several years	-	No	No
Patient 9 [2]	Between 47 and 62	F	-	Several years	-	No	No
Patient 10 [2]	Between 47 and 62	F	-	Several years	-	No	No
Patient 11 [2]	Between 47 and 62	F	-	Several years	-	No	No
Patient 12 [3]	54	F	Schizophrenia	10 years	Fluphenazine decanoate (25)	No	Yes
Patient 13 [25]	62	F	Schizophrenia	18 months	Mesoridazine (100) Clorazepate	No	No
Patient 14 [25]	68	F	Schizophrenia	10 years	Thioridazine (25)	No	Yes
Patient 15 [28]	63	F	Schizophrenia	Several	Fluphenazine decanoate Fluphenazine (20)	Yes	No
Patient 16 [28]	66	M	Bipolar disorder	8 days	Haloperidol (260)	Yes	No
Patient 17 [8]	42	F	Schizophrenia	16 months*	Haloperidol (90)	Yes	-
Patient 18 [8]	50	F	Schizophrenia	9 months*	Haloperidol (10)	No	-
Patient 19 [8]	57	M	Schizophrenia	7 months*	Haloperidol (8)	Yes	-
Patient 20 [8]	67	F	Bipolar disorder	8 months*	Haloperidol (8)	Yes	-
Patient 21 [8]	71	F	Korsakoff's syndrome	12 months*	Haloperidol (8) Chlorpromazine (150)	Yes	-
Patient 22 [8]	77	M	Mental retardatio	12 months *	Haloperidol (7)	Yes	-
Patient 23 [12]	52	M	Schizophrenia	20 years	-	No	No
Patient 24 [13]	75	M	Schizophrenia	30 years	Trifluoperazine (15) Fluphenazine decanoate (37.5)	No Just before	No
Patient 25 [7]	63	F	Schizophrenia	11 years	Haloperidol (8) Levopromazine (50) Lithium (600)	Yes	No
Patient 26 [7]	63	F	Schizophrenia	32 years	Propericiazine (50) Clocapramine (100)		Yes
Patient 27 [7]	44	F	Schizophrenia	13 years	Haloperidol (5) Propericiazine (60) Sulpiride (1200)	Yes	No
Patient 28 [7]	48	F	Schizophrenia	31 years	Haloperidol (5) Levopromazine (150) Sulpiride (1200)	Yes	No
Patient 29 [7]	58	M	Schizophrenia	11 years	Bromperidol (20) Sulpiride (600)	Yes	No
Patient 30 [10]	77	F	Schizophrenia	15 months	Thioridazine (75)	No	No
Patient 31 [4]	57	F	Acute paranoid episode	19 months	Haloperidol (30–50)	No	No
Patient 32 [29]	31	F	Schizophrenia	14 years	Haloperidol (5)	No	No
Patient 33 [26]	28	F	Schizophrenia	5 years	Zuclopentixol (200) Biperidene (4)	No	No
Patient 34 [19]	65	F	Major depression	10 years	Perphenazine (2) Amitriptyline (25) Paroxetine (20)	No	No

Patients identified by MEDLINE database search in December 2006.

**Table 2:** Newer Antipsychotics and the Rabbit Syndrome.

Patient	Age	Gender	Diagnosis	Drug (mg/day)	Duration of atypical therapy	Parkinsonian symptoms	Tardive dyskinasia
Patient 1 [30]	68	F	Schizophrenia	Risperidone (4)	4 months	No	No
Patient 2 [31]	27	M	Schizophrenia	Risperidone (4)	7 months	No	No
Patient 3 [32]	38	F	Schizophrenia	Risperidone (decresed from 2 to 1)	8 months	No	No
Patient 4 [33]	38	M	Major depression with psychotic features	Risperidone (4)	4 months	No	No
Patient 5 [34]	48	F	Schizophrenia	Clozapine (225)	7 months	No	Yes
Patient 6 [35]	29	F	Schizophreniform disorder	Aripiprazole (10)	6 weeks	Yes	No
Patient 7 [36]	56	M	Major depression with psychotic features	Risperidone (6) Biperiden (2)	4 months	Yes	No
Patient 8 [27]	22	M	Schizophrenia	Risperidone (4) Biperiden (4)	12 months	Yes	No
Patient 9 [37]	42	F	Bipolar I disorder	Risperidone (5)	3 weeks	No	No
Patient 10 [38]	74	-	Bipolar disorder	Olanzapine (20)	-	-	-
Patient 11 (our case)	50	F	Bipolar I disorder	Risperidone (decresed from 4 to 1 and discontinued)	3 months	Yes	No

### Etiopathogenesis

RS may appear during treatment with neuroleptic medications or after discontinuation of such medicines. The mechanisms triggering RS are believed to be similar to those involved in the pathogenesis of the neuroleptic-induced Parkinsonian syndrome [7]. In fact, RS may be due to a hypercholinergic state resulting from the neuroleptic blockade of dopaminergic neurons in the extrapyramidal system [12]. This would explain the response of RS to anticholinergic drugs [3,13].

Nishiyama [14], however, reported a case of RS that was treated successfully using haloperidol in a woman affected by multiple system atrophy. In this case, the etiopathogenetic mechanism of RS may be similar to TD, possibly suggesting a state of cholinergic hypofunction due to a denervation-type dopaminergic hypersensitivity in the basal ganglia [15].

Given that the disease mechanism of RS resembles the mechanism of parkinsonism and the mechanism of TD, it may be possible to attribute RS to an imbalance of the cholinergic and dopaminergic function in the basal ganglia. However, the etiopathogenesis of RS, parkinsonism, and TD, are still unclear.

Interestingly, neuroleptic exposure was found to be unrelated to following six documented cases of RS [11,16-18,20]. Truong [16] described a case of RS observed in a 76-year-old female who had undergone brain surgery 16 years earlier. Kamijo [17] reported a case related to ingestion of a massive quantity of phenol in a suicide attempt. Phenol intoxication may have caused RS by inducing cholinergic dominance in the central nervous system. RS was also observed in patients treated with imipramine, citalopram, paroxetine and methylphenidate [11,18-20]. It is possible that these antidepressants, potent inhibitors of serotonin reuptake, caused RS through a serotonin-mediated inhibition of dopaminergic neurotrasmission in the basal ganglia in predisposed subjects [21,22].

### Older antipsychotic medications and Rabbit Syndrome

Several case studies have been published on the relationship between older antipsychotics and RS (table 1). The use of high-potency neuroleptics with low anticholinergic activity, like haloperidol, is common to most of these cases. In fact, of the 34 cases reported in the literature, 12 were associated with haloperidol, and 8 with piperazinic phenotiazines (Fluphenazine, Perphenazine, Trifluoperazine). These high-potency drugs are known for a relative high tendency to induce parkinsonism [23]. This evidence, coupled with the fact that some patients who developed RS reported a previous history of the more usual parkinsonian side-effects [13], substantiates the hypothesis that RS and drug-induced parkinsonism may be mediated by a neuroleptic blockade of the same pool of dopaminergic neurons in the extrapyramidal system [3].

### Newer antipsychotic medications and the Rabbit Syndrome

To our knowledge, there are 10 reported cases of RS related to newer antypsychotics (table 2). Seven of the ten reported cases of RS are linked to risperidone. Risperidone is considered to be the atypical antipsychotic with the highest incidence of extrapyramidal symptoms [24]. This high incidence may be caused by the high affinity for the serotonin type 2 and dopamine type 2 receptors and the low affinity for cholinergic muscarinic receptors.

The three remaining cases occurred with clozapine, olanzapine and aripiprazole, respectively. These atypical antipsychotics are characterized by a relatively low incidence of extrapyramidal symptoms. It is interesting that, in the case induced by clozapine, TD preceded RS, which was developed after treatment for approximately 2 years with chlorpromazine, haloperidol, and fluphenazine decanoate, either in monotherapy or in combination.

We have observed a case of RS in a 50 year old white female. The patient was diagnosed with bipolar I disorder and was treated with lithium, gabapentin, lorazepam, risperidone, and citalopram. Though the intended treatment regimen was outpatient, the patient was hospitalized as a result of a severe depressive episode in which she was unable to care for herself physically. At the time of her admission, the dose of risperidone was 4 mg/day. Because of a shuffling gait, moderate drooling, and cog wheeling rigidity, the risperidone was first decreased to 1 mg and, after 1 day, it was completely discontinued. This led to a quick resolution of the extrapyramidal symptoms. No changes were made in her psychopharmacological treatment except for an adjustment of the lithium dose and the addition of trazodone. About 10 days after the discontinuation of risperidone, the patient developed involuntary movements of her mouth. The movements were fine, rhythmic, and rapid, along the vertical axis, and without lingual involvement. A diagnosis of risperidone withdrawal emergent RS was made. Risperidone was re-started at 1.5 mg/day. The symptoms noticeably decreased after a few hours and completely disappeared after 3 days. No extrapyramidal symptoms were observed.

### Treatment Options

Differently from TD, RS typically responds favorably to anticholinergic agents such as benztropine, biperiden, procyclidine and trihexyphenidyl [3]. RS typically disappears a few days after the introduction of an anticholinergic agent [4], but can, on occasion, reappear after stopping anticholinergic medications [12]. Researchers opted to implement a new treatment strategy after evidence was found that some patients with RS treated with an anticholinergic agent later developed TD [3,4,25]. The availability of the newer atypical antipsychotics has led to the implementation of additional treatment strategies.

Durst [26] successfully treated RS triggered by the typical neuroleptic zuclopenthixol by switching the patient from zuclopenthixol to olanzapine. After the administration of olanzapine (started at 5 mg/day and titrated up to a target dose of 10 mg/day), both the RS and the psychotic symptoms that the patient was presenting showed significant improvement. In another report, risperidone-induced RS not respondent to anticholinergic agents, disappeared after switching the patient to quetiapine. After 4 weeks of quetiapine treatment (starting dose of 100 mg/day and titration up to 700 mg/day) rabbit movements diminished significantly with further improvements during the follow-up period [27]. Switching to a new atypical antipsychotic with high anti-cholinergic properties, like olanzapine or clozapine, may be another possible treatment strategy for RS [24].

## Conclusion

Since Villeneuve first described RS in 1972 it has been associated with long-term exposure to the older neuroleptics (table 1). In the recent past RS was also observed in patients treated with the newer antypsychotics. To date, 11 cases, including the one documented in this paper, of RS have been reported in patients treated with atypical antipsychotics. Of these 11 cases, seven were related to the use or discontinuation of risperidone (table 2). The pharmacodynamics of risperidone may explain its tendency to induce RS. Although the possibility of RS should always be considered in patients treated with atypical neuroleptics in general, and with risperidone in particular, it is important to note that there is a low incidence of this syndrome.

RS can be successfully treated. RS responds well to treatment with anticholinergic agents, whereas TD would be worsened by such a treatment regimen. Conversely the use of dopamine depleters like reserpine, which have proven effective with TD, may worsen RS. Once again, this underscores the importance of distinguishing between these two syndromes. RS can be distinguished from typical oral dyskinesias because the movements observed in the latter are slower and less regular, involve the tongue, can be suppressed voluntarily by the patient, and do not persist during stage I non-REM sleep.

## Abbreviations

RS, rabbit syndrome

TD, Tardive dyskinesia

## Authors' contributions

MCDO conceived of the study and participated in its design and coordination. AF participated in the design of the study and gave the informations about the new reported case. FD and AC partecipated in the review of the literature and drafted the manuscript. AM partecipated in the review of the literature and drafted the manuscript. EF participated in the design of the study and helped to draft the manuscript. All authors read and approved the final manuscript.

## References

[B1] Villeneuve A The rabbit syndrome. A peculiar extrapyramidal reaction Can Psychiatr Assoc J 1972 17 2 SS69 SS72 5042912 10.1177/07067437720176s213

[B2] Jus K Jus A Gautier J Villeneuve A Pires P Pineau R Villeneuve R Studies on the action of certain pharmacological agents on tardive dyskinesia and on the rabbit syndrome Int J Clin Pharmacol 1974 9 138 145 4827556

[B3] Sovner R Dimascio A The effect of benztropine mesylate in the rabbit syndrome and tardive dyskinesia Am J Psychiatry 1977 134 1301 1302 910992 10.1176/ajp.134.11.1301

[B4] Schwartz M Weller B Erdreich M Sharf B Rabbit syndrome and tardive dyskinesia: two complications of chronic neuroleptic treatment J Clin Psychiatry 1995 56 212 7737962

[B5] Jus K Jus A Villeneuve A Villeneuve R Influence of concentration and motor performance on tardive dyskinesia and rabbit syndrome Can Psychiatr Assoc J 1973 18 327 330 4780751 10.1177/070674377301800412

[B6] Jus K Villeneuve A Jus A Tardive dyskinesia and the rabbit syndrome during wakefulness and sleep Am J Psychiatry 1972 129 765 4638729 10.1176/ajp.129.6.765

[B7] Wada Y Yamaguchi N The rabbit syndrome and antiparkinsonian medication in schizophrenic patients Neuropsychobiology 1992 25 149 152 1357583 10.1159/000118825

[B8] Yassa R Lal S Prevalence of the rabbit syndrome Am J Psychiatry 1986 143 656 657 2870650 10.1176/ajp.143.5.656

[B9] Jus K Jus A Villeneuve A Polygraphic profile of oral tardive dyskinesia and of rabbit syndrome: for quantitative and qualitative evaluation Diseases of the Nervous System 1973 34 27 32 4350828

[B10] Chiu HF Lam LC Chung DW Wing YK Shum PP Prevalence of the rabbit syndrome in Hong Kong J Nerv Ment Disord 1993 181 264 265 10.1097/00005053-199304000-00009 8097230

[B11] Fornazzari L Ichise M Remington G Smith I Rabbit syndrome, antidepressant use and cerebral perfusion SPECT scan findings J Psychiatr Neurosci 1991 16 4 227 229 PMC1188341 1786266

[B12] Deshmukh DK Joshi VS Agarwal MR Rabbit syndrome: a rare complication of long-term neuroleptic medication Br J Psychiatry 1990 157 293 1977486 10.1192/bjp.157.2.293

[B13] Almeida JH Neurolaptic side effects-The "rabbit syndrome" Int J Geriatr Psychiatry 1991 6 537 539 10.1002/gps.930060711

[B14] Nishiyama K Masuda N Kurisaki H A case of rabbit syndrome – its unique pharmacological features Rinsho Shinkeigaku 1993 33 663 665 8104749

[B15] Gerlach J Reisby N Randrup A Dopaminergic hypersensitivity and cholinergic hypofunction in the pathophysiology of tardive dyskinesia Psycopharmacol 1974 34 1 21 35 10.1007/BF00421217 4593518

[B16] Truong DD Hermanowicz N Mistura K Rabbit syndrome treated with botulin South Med J 1990 83 854 855 2371612 10.1097/00007611-199007000-00038

[B17] Kamijo Y Soma K Fukuda M Asari Y Ohwada T Rabbit syndrome following phenol ingestion J Toxicol Clin Toxicol 1999 37 509 511 10.1081/CLT-100102444 10465250

[B18] Parvin MM Swartz CM Dystonic rabbit syndrome from citalopram Clin Neuropharmacol 2005 28 289 291 10.1097/01.wnf.0000191348.10375.41 16340386

[B19] Gourzis P Argyriou AA Bakalidou C Beratis S Induction of the rabbit syndrome following coadministration of paroxetine, perphenazine, and amitriptyline Clin Neuropharmacol 2004 27 299 300 10.1097/01.wnf.0000150870.52253.fe 15613935

[B20] Mendhekar DN Duggal HS Methylphenidate-induced rabbit syndrome Ann Pharmacother 2006 40 2076 10.1345/aph.1H086 17047143

[B21] Leonard BE Pharmacological effects of serotonin reuptake inhibitors J Clin Psychiatry 1988 49 12 17 3045106

[B22] Kim SW Dysken MW Potential antidopaminergic effects of serotonin reuptake inhibitors J Clin Psychiatry 1991 52 42 1988420

[B23] Schwartz JT Brotman AW A clinical guide to antipsychotic drugs Drugs 1992 44 981 992 1282868 10.2165/00003495-199244060-00007

[B24] Tarsy D Baldessarini RJ Tarazi FI Effects of newer antipsychotic on extrapyiramidal function CNS Drugs 2002 16 23 45 10.2165/00023210-200216010-00003 11772117

[B25] Weiss KJ Ciraulo DA Shader RI Physostigmine test in the rabbit syndrome and tardive dyskinesia Am J Psychiatry 1980 137 627 628 6102848 10.1176/ajp.137.5.627

[B26] Durst R Katz G Zislin J Raskin S Kalman I Rabbit syndrome treated with olanzapine Br J Psychiatry 2000 176 193 10.1192/bjp.176.2.193 10755060

[B27] Altindag A Yanik M A case of rabbit sindrome treated with quetiapine Eur Psychiatry 2005 20 574 575 10.1016/j.eurpsy.2004.11.014 16337895

[B28] Todd R Lippman S Manshadi M Chang A Recognition and treatment of rabbit syndrome, an uncommon complication of neuroleptic terapies Am J Psychiatry 1983 140 1519 1520 6137964 10.1176/ajp.140.11.1519

[B29] Damodaran SS Thankamma JK Reversal of rabbit syndrome with olanzapine Aust N Z J Psychiatry 2000 34 172 173 11185940 10.1080/000486700377

[B30] Schwartz M Beny A Sharf B Risperidone-induced rabbit syndrome Br J Psychiatry 1998 173 267 268 9926106 10.1192/bjp.173.3.267b

[B31] Levin T Heresco-Levy U Risperidone-induced rabbit syndrome: an unusual movement disorder caused by an atypical antipsychotic Eur Neuropsycopharmacol 1999 9 137 139 10.1016/S0924-977X(98)00016-9 10082239

[B32] Nashimura K Tsuka M Horikawa N Withdrawal-emergent rabbit syndrome during dose reduction of risperidone Eur Neuropsycopharmacol 2001 11 4 323 324 10.1016/S0924-977X(01)00095-5 11532387

[B33] Hoy JS Alexander B Rabbit syndrome secondary to risperidone Pharmacotherapy 2002 22 513 515 10.1592/phco.22.7.513.33669 11939686

[B34] Sethi S Bhargava SC Clozapine-induced rabbit syndrome J Clin Psychiatry 2003 64 219 12633136 10.4088/jcp.v64n0215e

[B35] Mendhekar DN Aripiprazole-induced rabbit syndrome Aust N Z J Psychiatry 2004 38 561 10.1111/j.1440-1614.2004.01412.x 15255832

[B36] Eren I Ozcankaya R Altinyazar V Risperidone-induced rabbit syndrome in mood disorder Eur Psychiatry 2004 19 452 453 10.1016/j.eurpsy.2004.06.022 15504657

[B37] Mendhekar DN Rabbit Syndrome induced by combined lithium and risperidone Can J Psychiatry 2005 50 369 15999955 10.1177/070674370505000618

[B38] Sabolek M Bayerle M Rabbit syndrome due to olanzapine Psychiatr Prax 2005 32 202 204 10.1055/s-2004-834759 15852213

